# Mammotome-assisted removal with minimal incision of large juvenile fibroadenoma of breast: A case report

**DOI:** 10.1097/MD.0000000000019442

**Published:** 2020-03-06

**Authors:** Tongling Wang, Lin Zhu

**Affiliations:** Department of General Surgery, The Fourth Affiliated Hospital of Zhejiang University School of Medicine, Yiwu, Zhejiang, China.

**Keywords:** cosmetic surgery, giant juvenile fibroadenoma, Mammotome-combined excision

## Abstract

**Introduction:**

Giant juvenile breast fibroadenoma can cause deformity and should be excised. Cosmesis is an important consideration in young patients.

**Patient concerns:**

The patient was admitted to our hospital for a mass of 6 × 6 cm in her left breast.

**Diagnose:**

A giant juvenile fibroadenoma.

**Interventions:**

With the help of Mammotome, we translated the larger mass to smaller one and removed it via a small circumareolar incision with no residual tumor.

**Outcomes:**

There was no recurrence of disease after 2 years.

**Conclusion:**

Mammotome-combined resection is a new approach that can be used to excise giant fibroadenomas with a minimal incision, and provides a favorable contour to the breast.

## 1 Introduction

An ultrasound-guided Mammotome breast biopsy system (Ethicon EndoSurgery, Inc., Cincinnati, OH) could be used not only as a valuable tool for investigation of suspicious breast lesions, but also therapeutically for the complete removal of benign breasts lumps, such as fibroadenomas. Contrasting with surgical excision, it is minimally invasive and can be performed under local anesthesia without cosmetic impairment and problematic breast tissue scars.^[1,2]^ Its additional therapeutic potential is dependent on the diameter of the benign lesion. Lumps <3 cm generally can be completely removed. Using this technique, the cosmetic outcome is better than that of traditional open surgery. It is rarely used in fibroadenomas over 5 cm with a high risk of residual tumor increasing in size.^[3]^ Here, we report excision of a giant juvenile breast fibroadenoma of 6 cm with only a 3-cm circumareloar incision in combination with Mammotome.

## 2 Case report

**Figure 1:**
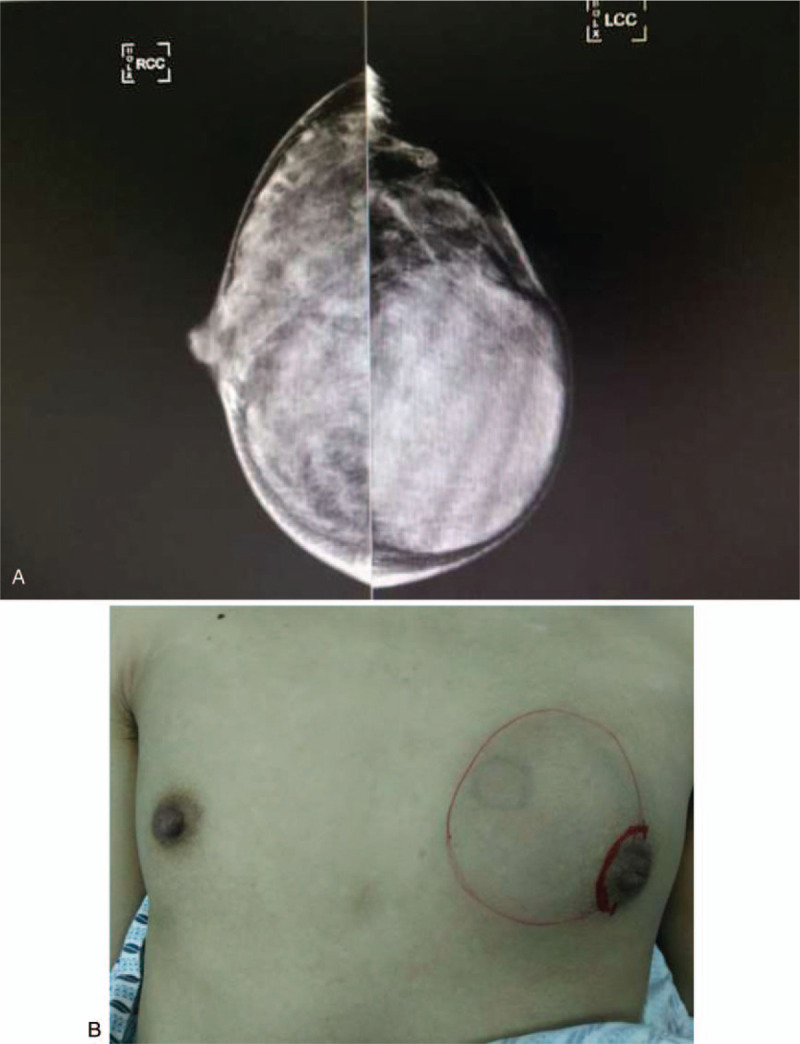
(A) Preoperative mammogram demonstrating the lesion of left breast. (B) Preoperative photo depicting the large mass causing visible significant asymmetry and the nipple shift.

A 19-year-old Chinese adolescent complained that her left breast was larger than the right breast and she had been able to feel a mass in her left breast for 2 months. She was admitted to our hospital on February 19, 2017, with no history of trauma, nipple discharge, fever, or weight loss. On physical examination, enlargement of the left breast with one palpable mass was observed (Fig. 1). The mass was about 6 × 6 cm, movable, solitary, firm, and well defined. It was not tender and did not adhere to the skin or the underlying structures. The local temperature of the overlying skin was normal. There was no axillary lymphadenopathy.

Breast ultrasound demonstrated a homogenous, hypoechoic solid mass with no phyllodes. Mammography showed a large mass with high density and well definition (Fig. 1). Based on the clinical and experimental findings, the mass was considered a giant fibroadenoma. Due to the history of progressive enlargement of the left-sided lesion, she underwent an excision biopsy instead of needle biopsy. To avoid a disfiguring scar, we planned to use the 8-gauge Mammotome to shrink the mass and then perform the lumpectomy through a tiny circumareolar incision.

**Figure 2:**
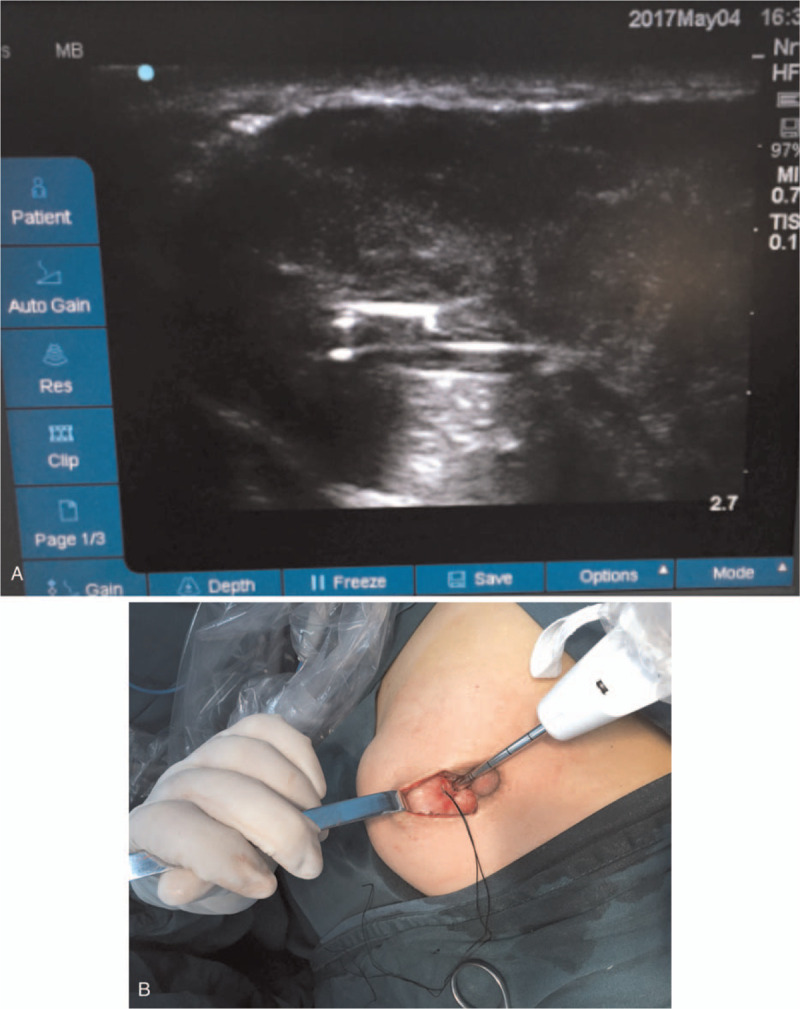
(A) Intraoperative ultrasound showing the needle been put into the center of the mass. (B) Intraoperative photo taken immediately after the needle been placed into the center of the mass with a purse string suture.

**Figure 3:**
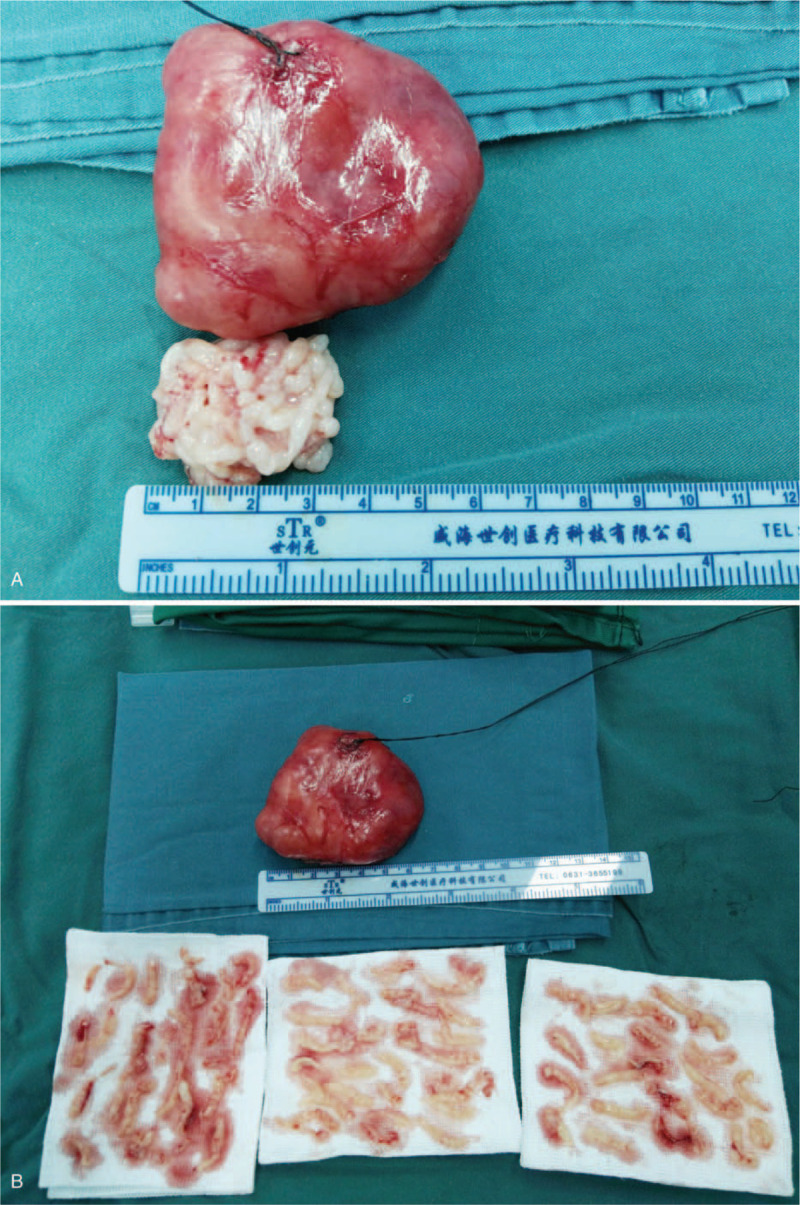
(A) Specimen of tumor mass measuring 6 cm. (B) Intact specimen removed from the incision and fragmentary specimens removed from the Mammotome.

Local anesthesia (200 mg of 2% lidocaine, 75 mg of ropivacaine, 0.5 mg of 1:1000 epinephrine, and 20 mL of sodium chloride injection) was given in the operation area. A 3-cm circumareolar incision was made, which allowed the surface of the tumor to be separated. Under ultrasound monitoring, the tip of 8 G Mammotome needle was placed in the center of the tumor. We first removed the inner tissue to reduce the tumor to an appropriate size and avoided to cut through the capsule (Fig. 2). A purse string suture was made to close the portal of the Mammotome needle. After the inner tissue was removed, the diameter of the tumor was reduced to about 3 cm. Then, the tumor could be completely removed through the incision completely (Fig. 3). There was no residual tumor remains. The operation time was <60 minutes and the mean blood loss was about 5 mL.

**Figure 4:**
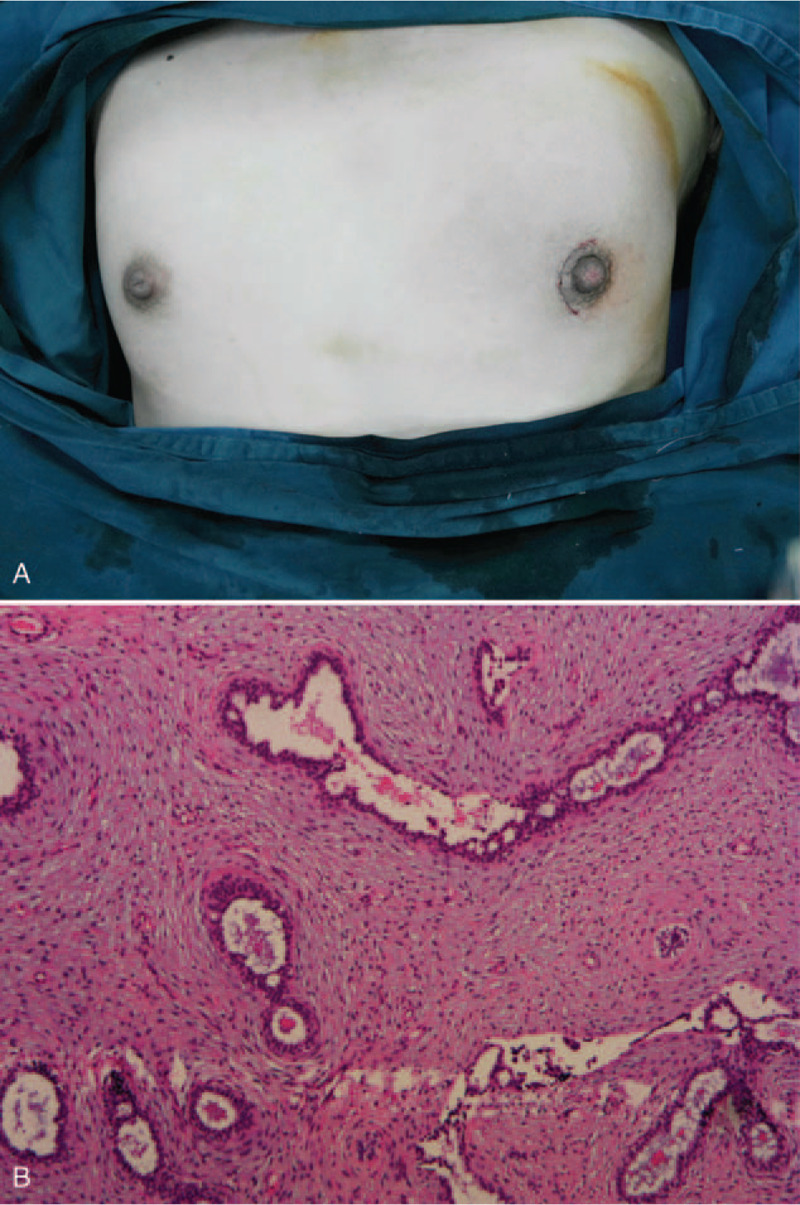
(A) Perioperative photo taken immediately after closing the incision showing an excellent cosmetic outcome. (B) The appearance of ductal and stromal elements of the juvenile fibroadenoma (hematoxylin and eosin, original magnification ×10).

**Figure 5:**
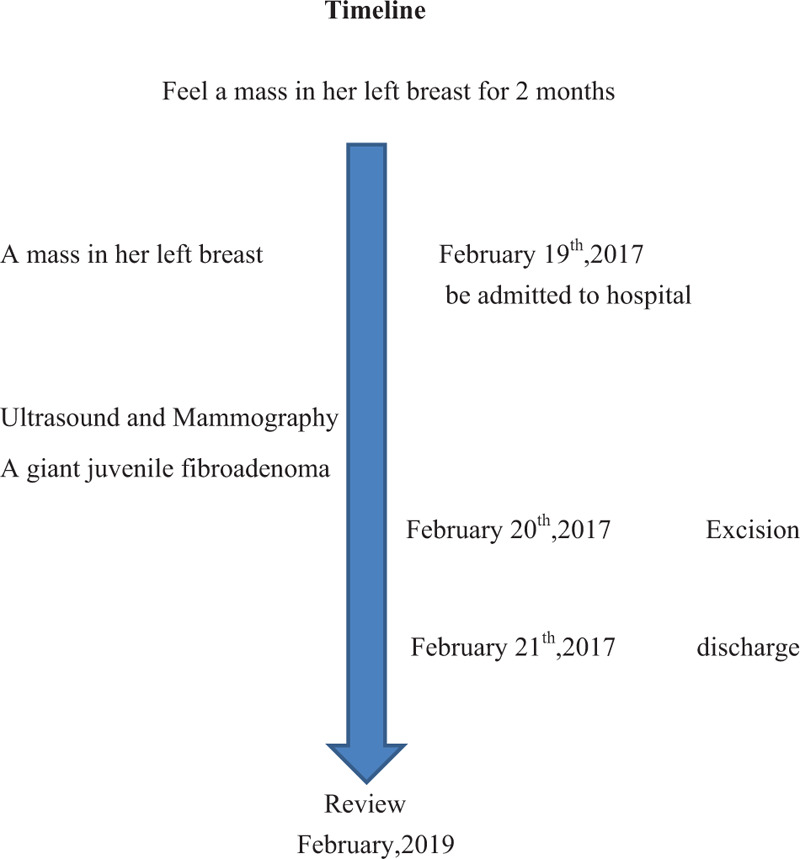
Timeline.

Postoperatively, there was no pain, hemorrhage, or hematoma formation, and the patient's recovery was uneventful. The specimen was submitted to the pathology department, and the diagnosis of juvenile fibroadenoma was confirmed (Fig. 4). The patient returned to the clinic 2 years later, with good cosmetic results and no recurrence (Fig. 5).

## 3 Discussion

Breast lumps in adolescents are mostly benign in nature. Juvenile fibroadenoma is the most common, comprising nearly 75% of breast masses in puberty.^[4]^ Fibroadenoma is a benign tumor that may grow large in size and distort the shape of the breast. Giant juvenile fibroadenoma, defined as >5 cm, >500 g, or replacing at least four-fifths of the breast, is very rare. Due to its rapid growth and immense size at the time of presentation, it can strain and distort the shape of the affected breast.^[5]^ Unlike smaller fibroadenomas, which can be treated by other means, resection of giant juvenile fibroadenomas is required as a diagnostic and therapeutic approach.^[6]^ Varying techniques in surgical extirpation have been described in order to optimize esthetics and minimize distortion. These include video-assisted endoscopic extirpation with and without morcellation,^[7]^ “Swiss-roll” technique,^[8]^ “Saw Tooth” operation,^[9]^ circumareolar incision with T-shaped or lateral extension,^[10]^ a sub-mammary incision,^[11]^ endovascular embolization before operation,^[12]^ reduction mammaplasty, and mastectomy.^[13]^ A breast incision should be performed after taking into account not only the standard of care for giant juvenile fibroadenomas with preservation of maximal breast tissue but also the cosmesis. Breast tumor surgery is often performed with the patient under local anesthesia. However, in the case of large tumors, surgery is usually performed with the patient under general anesthesia.^[14]^ Due to these concerns, we tried to find a method that we could use to excise a giant mass completely using only a short incision and local anesthesia.

Due to its minimally invasive and highly precise nature, the Mammotome has proven effective for the treatment of benign breast lesions. But it has shown a high risk of residual tumor increasing in size and it is rarely used in fibroadenomas over 5 cm.^[3]^ Using the Mammotome device innovatively we translate a giant juvenile fibroadenoma to smaller one. The inside of the breast mass was removed using the Mammotome to reduce the size of the large mass. It then became possible to be completely removed through a 3-cm circumareolar incision, keeping its morphology intact, like smaller fibroadenomas. There is no risk of leaving fragments behind. This method can be performed with the patient under local anesthesia in <60 minutes. The pain was controlled well. The patient was satisfied with the small scar and cosmetic results. The Mammotome can be used in combination with all kinds of incisions, so the innovate technique may be used for giant masses located in all quadrants of the breast, especially for those with regular shape and complete capsule.

The size of the tumor and age and stage of sexual maturity of the patient should be taken into account to select the most appropriate surgical approach.^[8]^ The breasts of most Asians are smaller than white and black patients. Breast masses >10 cm are rare. We should choose an appropriate surgical procedure for large breast masses in adolescents to preserve the normal breast tissue with strong cosmesis and preservation of lactation function. Mammotome-assisted resection described here may be a good choice for these giant juvenile fibroadenoma of 5 to 10 cm, especially for Asian patients.

## Acknowledgments

The authors thank LetPub (www.letpub.com) for its linguistic assistance during the preparation of this manuscript.

## Author contributions

**Investigation:** Tongling Wang.

**Methodology:** Tongling Wang, Lin Zhu.

**Project administration:** Tongling Wang, Lin Zhu.

**Resources:** Lin Zhu.

**Writing – original draft:** Tongling Wang.

**Writing – review & editing:** Tongling Wang, Lin Zhu.
